# Pharmacological profiling of zebrafish behavior using chemical and genetic classification of sleep-wake modifiers

**DOI:** 10.3389/fphar.2015.00257

**Published:** 2015-11-03

**Authors:** Yuhei Nishimura, Shiko Okabe, Shota Sasagawa, Soichiro Murakami, Yoshifumi Ashikawa, Mizuki Yuge, Koki Kawaguchi, Reiko Kawase, Toshio Tanaka

**Affiliations:** ^1^Department of Molecular and Cellular Pharmacology, Pharmacogenomics and Pharmacoinformatics, Mie University Graduate School of MedicineTsu, Japan; ^2^Mie University Medical Zebrafish Research CenterTsu, Japan; ^3^Department of Systems Pharmacology, Mie University Graduate School of MedicineTsu, Japan; ^4^Department of Omics Medicine, Mie University Industrial Technology Innovation InstituteTsu, Japan; ^5^Department of Bioinformatics, Mie University Life Science Research CenterTsu, Japan

**Keywords:** zebrafish, behavior, profiling, hypnotics, psychostimulants, hypocretin

## Abstract

Sleep-wake states are impaired in various neurological disorders. Impairment of sleep-wake states can be an early condition that exacerbates these disorders. Therefore, treating sleep-wake dysfunction may prevent or slow the development of these diseases. Although many gene products are likely to be involved in the sleep-wake disturbance, hypnotics and psychostimulants clinically used are limited in terms of their mode of action and are not without side effects. Therefore, there is a growing demand for developing new hypnotics and psychostimulants with high efficacy and few side effects. Toward this end, animal models are indispensable for use in genetic and chemical screens to identify sleep-wake modifiers. As a proof-of-concept study, we performed behavioral profiling of zebrafish treated with chemical and genetic sleep-wake modifiers. We were able to demonstrate that behavioral profiling of zebrafish treated with hypnotics or psychostimulants from 9 to 10 days post-fertilization was sufficient to identify drugs with specific modes of action. We were also able to identify behavioral endpoints distinguishing GABA-A modulators and hypocretin (hcrt) receptor antagonists and between sympathomimetic and non-sympathomimetic psychostimulants. This behavioral profiling can serve to identify genes related to sleep-wake disturbance associated with various neuropsychiatric diseases and novel therapeutic compounds for insomnia and excessive daytime sleep with fewer adverse side effects.

## Introduction

Sleep-wake states are impaired in various neurological and neuropsychiatric disorders, including AD (Coogan et al., 2013; Musiek et al., 2015; Peter-Derex et al., 2015), Parkinson’s disease (Christensen et al., 2015; Latreille et al., 2015), depression (Saltiel and Silvershein, 2015), schizophrenia (Dell’Osso et al., 2014), and autism spectrum disorders (ASDs; Hollway and Aman, 2011). For example, AD patients exhibit disturbances in the timing and duration of the sleep cycle, primarily manifested as increased wakefulness at night and increased sleep during the day. Poor sleep quality can be an early sign of cognitive decline (Potvin et al., 2012). It has been suggested that tangle formation in the suprachiasmatic nucleus and cholinergic cells in basal forebrain may occur at early stages of AD, which may lead to circadian rhythm disruption in AD (Coogan et al., 2013). Furthermore, the sleep-wake disruption appears to increase the levels of amyloid plaques and tau aggregation in the brain (Rothman et al., 2013; Musiek et al., 2015). Therefore, treating sleep-wake dysfunction might prevent or slow the development of subsequent AD pathology and later dementia (Musiek et al., 2015). It has also been demonstrated that children with more severe cases of ASD are more likely to develop sleep disorders (Hollway and Aman, 2011). The severity of ASD symptom (i.e., deficits in social communication and social interaction and restricted/repetitive behaviors) may serve as vulnerability factors and predispose children with ASD to insomnia when presented with environmental stressors such as unpredictability in the environment and changes in their routines (Hollway and Aman, 2011).

Addressing the primary cause underlying each disease may be enough to counter the sleep-awake disturbance. However, if patients continue to have sleep-awake disturbance, hypnotics, and psychostimulants are considered for the treatment of insomnia and excessive daytime sleep, respectively. Hypnotics form a large group, including benzodiazepines and non-benzodiazepines. Both benzodiazepines and non-benzodiazepines interact with the same allosteric benzodiazepine-binding site on GABA-A receptors to increase their activity (Ramirez et al., 2013). They decrease sleep latency (Mitchell and Weinshenker, 2010). However, they have side effects including cognitive and psychomotor impairment, rebound insomnia, anterograde amnesia, and increased risk of motor collisions and falls (Equihua et al., 2013). The impairment observed across benzodiazepine and non-benzodiazepine compounds may be a result of the widespread expression of GABA-A receptors throughout the brain (Equihua et al., 2013; Ramirez et al., 2013). Psychostimulants consists of sympathomimetic psychostimulants and non-sympathomimetic psychostimulants (Banerjee et al., 2004). Sympathomimetic psychostimulants promote wakefulness by enhancing monoaminergic transmission by increasing the release and inhibiting the reuptake of these neurotransmitters (Banerjee et al., 2004; Sinita and Coghill, 2014). Non-sympathomimetic psychostimulants promote wakefulness in the absence of the other arousing effects typically seen with the sympathomimetic drugs (Banerjee et al., 2004; Mitchell and Weinshenker, 2010). Although the effects of sympathomimetic psychostimulants on wakefulness are stronger than non-sympathomimetic psychostimulants, sympathomimetic psychostimulants have side effects, including excess locomotor activities (Kim, 2012), sleep rebound (Schwartz, 2009), and high abuse liability (Anderson et al., 2012). Therefore, there are growing demands for developing new hypnotics and psychostimulants with high efficacy and a low profile for side effects (Urban and Gao, 2014; Dubey et al., 2015). To develop these novel hypnotics and psychostimulants, the use of animal models is indispensable. Although rodents have been the model of choice, screening many compounds using rodents has several drawbacks including costs and ethical considerations. Using alternative non-mammalian animal models may relieve some of these pressures by allowing testing of large numbers of subjects while reducing expenses and minimizing the use of mammalian subjects (Nishimura et al., 2015).

Zebrafish use all neurotransmitters currently known to be important for the regulation of sleep and wakefulness (Panula et al., 2010; Chiu and Prober, 2013; Elbaz et al., 2013). Like humans, zebrafish are diurnal and thus exhibit peak activity during the light phase and increased quiescence during the dark phase (Sorribes et al., 2013). It has been demonstrated that zebrafish exhibit behaviors characteristic of sleep, including having a quiescent state regulated by a circadian rhythm, reduced sensory responsiveness, and homeostatic regulation (Zhdanova, 2006; Chiu and Prober, 2013; Sigurgeirsson et al., 2013). It has also been demonstrated that the sleep-awake state of zebrafish can be assessed using the swimming velocity (Zhdanova, 2006; Chiu and Prober, 2013; Sigurgeirsson et al., 2013). Behavioral assays in zebrafish have also been successfully used to assess changes in sleep-awake states in response to pharmacological and genetic interventions (Prober et al., 2006; Rihel et al., 2010; Sigurgeirsson et al., 2011). However, there have been no studies comparing the behavior of zebrafish treated with clinically used hypnotics and psychostimulants.

The purpose of this study was to examine whether profiling of zebrafish behavior could be used to classify sleep-wake modifiers corresponding to their mode of action. We analyzed the number of transition between rest and active states and the percentage in the rest state based on their swimming velocity. We also analyzed 12 behavioral endpoints comprised of four phenotypes (distance moved, distance to center zone, frequency entering center zone, and turn angle) at three mobility states (high, medium, and low mobility). We were able to demonstrate that the behavioral profiling of zebrafish classified sleep-wake modifiers based on their specific modes of action, suggesting that zebrafish may be a useful tool to analyze the effects of chemicals and genes on sleep-wake states and to develop novel hypnotics and psychostimulants.

## Materials and Methods

### Compounds

Triazolam, ZPD, MPD, and MDF were purchased from Sigma (St. Louis, MO, USA). TCS-1102 was purchased from Tocris (Bristol, UK). PML was purchased from Tokyo Kasei (Tokyo, Japan). These compounds were dissolved in dimethyl sulfoxide (Nacalai, Kyoto, Japan) to make stock solutions.

### Zebrafish Husbandry

Zebrafish (AB line from ZIRC) were maintained according to the methods described by Westerfield (Westerfield, 2007) with some modification. Briefly, zebrafish were raised at 28.5 ± 0.5°C with a 14-h/10-h light/dark cycle. Embryos were obtained via natural mating and cultured in 0.3× Danieau’s solution [19.3 mM NaCl, 0.23 mM KCl, 0.13 mM MgSO_4_, 0.2 mM Ca(NO_3_)_2_, 1.7 mM HEPES, pH 7.2] until 10 days post-fertilization (dpf).

### Generation of Hypocretin-KO Zebrafish

Transcription activator-like effector nucleases targeting exon 2 of the zebrafish hcrt gene were constructed using the Golden Gate TALEN and TAL Effector Kit 2.0 (Addgene #1000000024; Cermak et al., 2011) and YAMAMOTO Lab TALEN Accessory Pack (Addgene #1000000030; Sakuma et al., 2013). Briefly, single DNA-binding repeats were assembled into intermediate array vectors. The assembled repeat arrays were subsequently inserted into the final destination vectors, pCS2TAL3-DD and pCS2TAL3-RR (Addgene #37275 and #37276; Dahlem et al., 2012).

The mRNA of TALEN was synthesized using mMessage mMachine SP6 Kit (Life Technologies, Carlsbad, CA, USA). The TALEN mRNAs (300 ng/mL each) were injected into two to eight cell-stage embryos of zebrafish. After injection, the embryo were cultured in 0.3× Danieau’s solution until 5 dpf and reared on an artificial diet (Meito Suien, Nagoya, Japan) and *Artemia* (Kitamura, Kyoto, Japan) at 28.5°C under a 14-h light/10-h dark period.

At 4 months post-fertilization (mpf), genomic DNA was extracted from the F0 fins according to a previous report (Ota et al., 2013). To detect TALEN-induced mutations, a short fragment of the hcrt gene that included the target site was amplified from genomic DNA using primers (5′-gtctcccaacagaagctcca-3′ and 5′-cccactttacgtttgccaag-3′). Three-step PCR was carried out: 45 cycles of 94°C for 30 s, 60°C for 30 s, and 68°C for 30 s. The PCR products were electrophoresed on 10% poly-acrylamide gels. The F0 fish in which the TALEN-induced mutation was detected were crossed with the AB strain to obtain F1 progeny. The F1 generation was reared and the mutation was examined as described above. The PCR amplicons were cloned into a pGEM-T Easy vector (Promega, Madison, WI, USA) and the sequences were analyzed using the M13 forward primer. An F1 female zebrafish and an F1 male zebrafish having the same mutations in the hcrt gene were crossed to obtain F2 progeny. The F2 progeny were used for the behavior analysis. After the behavior analysis, genomic DNA was extracted from each zebrafish and the genotype and the sequence were examined as described above.

### Behavior Analysis

An overview of the behavior analysis in this study is shown in Supplementary Figure S1. The behavioral test was performed during the same time frame. Eighty-four zebrafish at 7 or 9 dpf were placed individually into wells on a round 48-well plate (10 mm diameter, 300 μL of 0.3× Danieau’s solution) at 1 pm. The 48-well plate was placed in an incubator at 28.5°C with constant light (255 lx) from 1 to 3 pm. Then, the 48-well plate was placed in Daniovision (Noldus, Wageningen, The Netherlands), which was blocked from daylight and illuminated from below with white light (255 lx) from 3 to 5 pm. The behavior of zebrafish in each well was monitored by Daniovision with a resolution of 1024 × 768 pixels at 25 frames per seconds (fps). After the first monitoring, the 48-well plate was placed in an incubator at 28.5°C with constant light (255 lx). At 6 pm, 300 μL of 0.3× Danieau’s solution with or without compounds were added to each well of the 48-well plate. Then, the 48-well plate was placed in Daniovision and the behavior of zebrafish was monitored with a resolution of 1024 × 768 pixels at 25 fps. In the second monitoring, the light (255 lx) was turned on from 6 to 9 pm (Zeitgeber time, ZT 0-3) and 7 am to 6 pm (ZT 13-24). Eight larva were assigned to examine the effect of each concentration of the compound. Two independent experiments were performed for each compound.

The recorded video images were subjected to Ethovision XT11 (Noldus) to measure the behavior of zebrafish in each well. For the first monitoring, total distance moved and turn angle were measured. Turn angle represents the change in direction of the center point of the animal between two consecutive samples. If the distance moved and turn angle of zebrafish showed greater than two standard deviations from the median of the 48 samples, the zebrafish was excluded from further analysis. For the second monitoring, the mean velocity for each 6 s, total distance moved, distance to center zone (2 mm radius circle) of the well, frequency entering the center zone, turn angle, and mobility were measured. Mobility is calculated by taking every pixel identified as the subject and comparing it between the current image and the previous one. If all the pixels are the same, there is zero mobility. If all the pixels are different, there is 100% mobility. In this study, we defined 5–35, 35–65, and 65–95% as low, medium, and high mobility.

Velocity data were exported from Ethovision to a text edit file and imported into a custom-made program using R. The data were divided into 6-s bouts (comprising 150 measurements each). If 10 or more successive bouts (equal to or over 60 s) had a mean velocity for each bout below a defined threshold, it was designated as rest state (Supplementary Figure S2). In contrast, if the mean velocity of a bout was equal to or over the threshold among 10 successive bouts, it was designated as active state. We also measured the number of transition between the rest and active states. The percentage in the rest state and the number of transition between rest and active states were calculated for each hour or each period (L1; ZT 0-3, D; ZT 3-13, L2; ZT 13-24). We analyzed the velocity data changing the threshold. When we used 0.5 mm/s as the threshold, the percentage in the rest state during dark period was significantly higher than those during light periods. However, the number of transition between rest and active states during dark period was not significantly higher than those during light periods, suggesting that using 0.5 mm/s as the threshold may be too stringent. When we used 0.2 mm/s as the threshold, both the number of transition between rest and active state and the percentage in the rest state during dark period were significantly higher than those during light periods, which is consistent to previous studies using zebrafish (Yokogawa et al., 2007; Elbaz et al., 2012). Therefore, we decided to use 0.2 mm/s as the threshold in this study.

The data for total distance moved, the mean distance to the center zone, the frequency entering the center zone, and the mean absolute turn angle for each period (L1, D, and L2) were also measured for the time showing high, medium, or low mobility. The means were compared by analysis of variance using Prism 6 (Graphpad, La Jolla, CA, USA). Alpha was set at 0.05 and the Dunnett’s multiple comparisons test was used for *post hoc* analyses when significant effects were found.

For HCL and PCA, the data for each behavioral endpoint were normalized to the controls and constituted a feature vector. HCL was performed with the Multiexperiment Viewer (Howe et al., 2011) using the Covariance value with average linkage as the metric. PCA was performed using Bioconductor (Gentleman et al., 2004) and “rgl” package (Adler et al., 2014).

## Results

### ZPD was more Effective at 9 dpf than 7 dpf

It has been demonstrated that rest-like behavior in zebrafish can be defined as the duration of non-movement and that rest-like behavior can be used as a measure of sleep states (Rihel et al., 2010; Sigurgeirsson et al., 2011). Therefore, we first assessed the effects of clinically used hypnotics on the number of transitions between rest and active states and the percentage in the rest states observed in zebrafish. An overview of the behavior analysis is shown in Supplementary Figures S1 and S2, and the experimental detail is described in the methods section.

**FIGURE 1:**
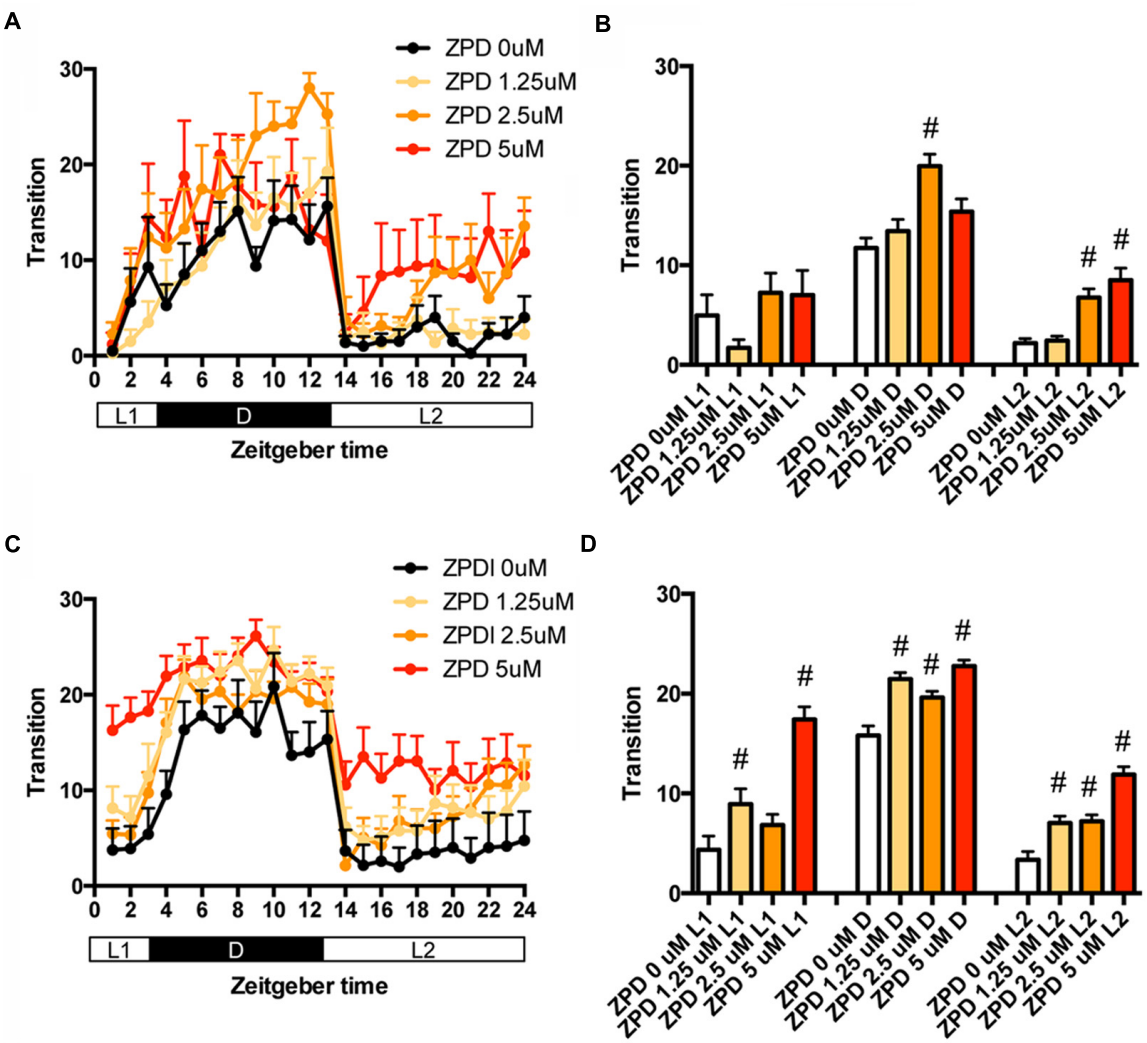
**Effects of zolpidem on the transition between rest and active states of zebrafish from 7 to 8 dpf and 9 to 10 dpf.** The number of transition states of zebrafish treated with zolpidem from 7 to 8 dpf **(A)** and from 9 to 10 dpf **(C)** are shown for each hour. The number of transitions of zebrafish treated with zolpidem from 7 to 8 dpf **(B)** and from 9 to 10 dpf **(D)** are shown during L1 (ZT 0-3), D (ZT 3-13), and L2 (ZT 13-24). The data are represented as means with the standard error of mean. #*p* < 0.05 vs. control.

We first examined the effects of ZPD, one of the most widely prescribed hypnotics, on the transition between rest and active states of zebrafish from 7 to 8 dpf. The transition was significantly increased by ZPD in D (2.5 μM) and L2 (2.5 and 5 μM) but not in L1 (**Figures 1A,B** and Supplementary Table S1). We next examined the effects of ZPD on the transition of zebrafish from 9 to 10 dpf. As shown in **Figures 1C,D**, the transition was significantly increased by ZPD in L1, D and L2 at 1.25 μM (**Figures 1C,D** and Supplementary Table S1).

**FIGURE 2:**
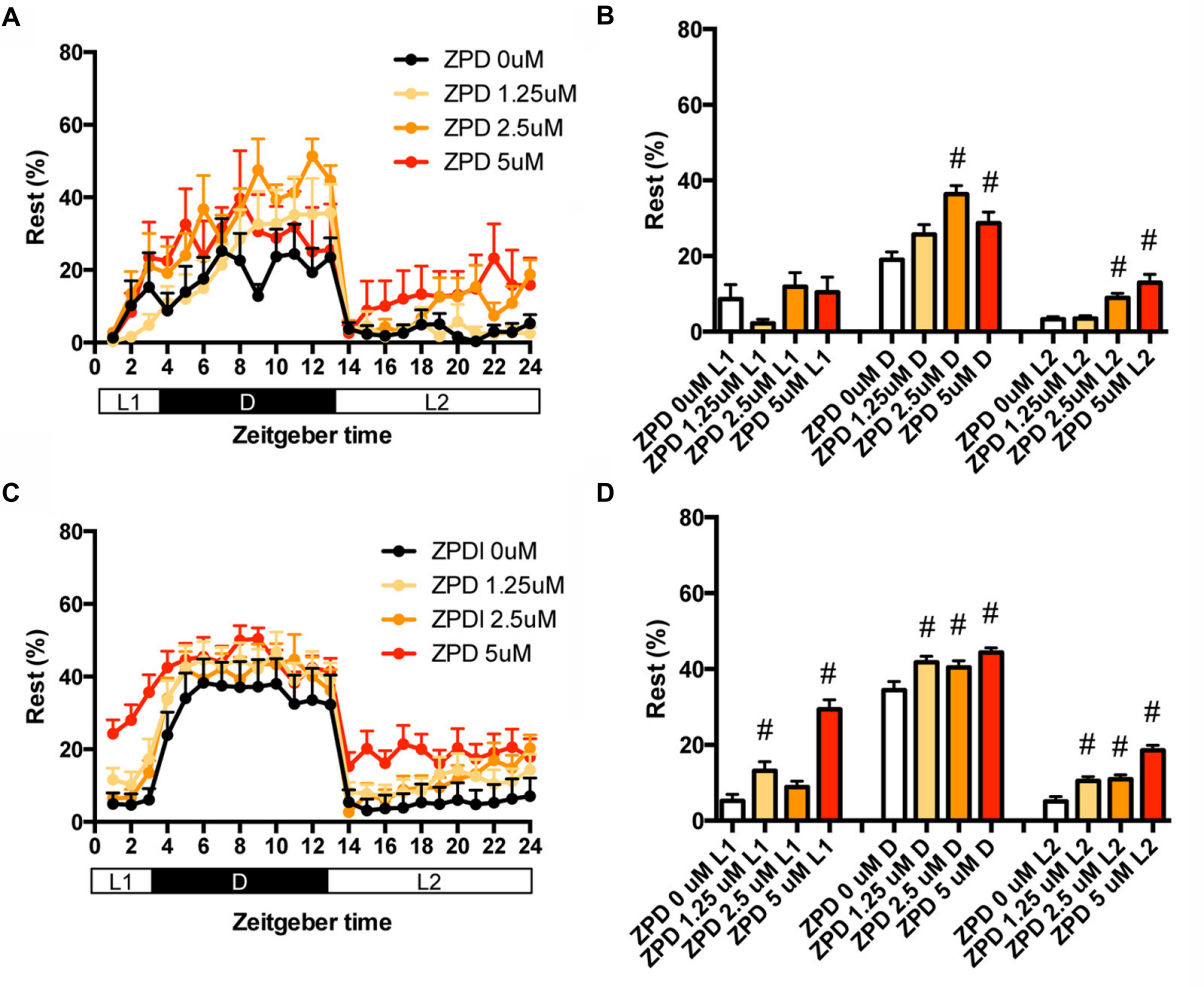
**Effects of zolpidem on the rest state of zebrafish from 7 to 8 dpf and 9 to 10 dpf.** The rest state of zebrafish treated with zolpidem from 7 to 8 dpf **(A)** and from 9 to 10 dpf **(C)** are indicated as percentage of total behavior during each hour. The rest state of zebrafish treated with zolpidem from 7 to 8 dpf **(B)** and from 9 to 10 dpf **(D)** are shown as percentage in L1 (ZT 0-3), D (ZT 3-13), and L2 (ZT 13-24). The data are represented as means with the standard error of mean. #*p* < 0.05 vs. control.

We then examined the effects of ZPD on the percentage in the rest state of zebrafish at between 7 and 8 dpf (**Figures 2A,B** and Supplementary Table S2) and between 9 and 10 dpf (**Figures 2C,D** and Supplementary Table S2). The effects of ZPD were the same as those on the transition between rest and active states.

These results suggest that ZPD may increase the percentage in the rest state by decreasing the duration of the wake state in zebrafish (Supplementary Figure S2C, Pattern 2), which is similar to the effect of ZPD in mammals (Greenblatt and Roth, 2012; Berdyyeva et al., 2014). These results are also consistent with previous results showing the increase of sensitivity to ZPD during development in rats (Wall, 2005; Chudomel et al., 2009). Therefore, we decided to examine the effects of sleep/awake modifiers on zebrafish from 9 to 10 dpf.

### Hypnotics and hcrt-KO Increased the Rest State of Zebrafish

**FIGURE 3:**
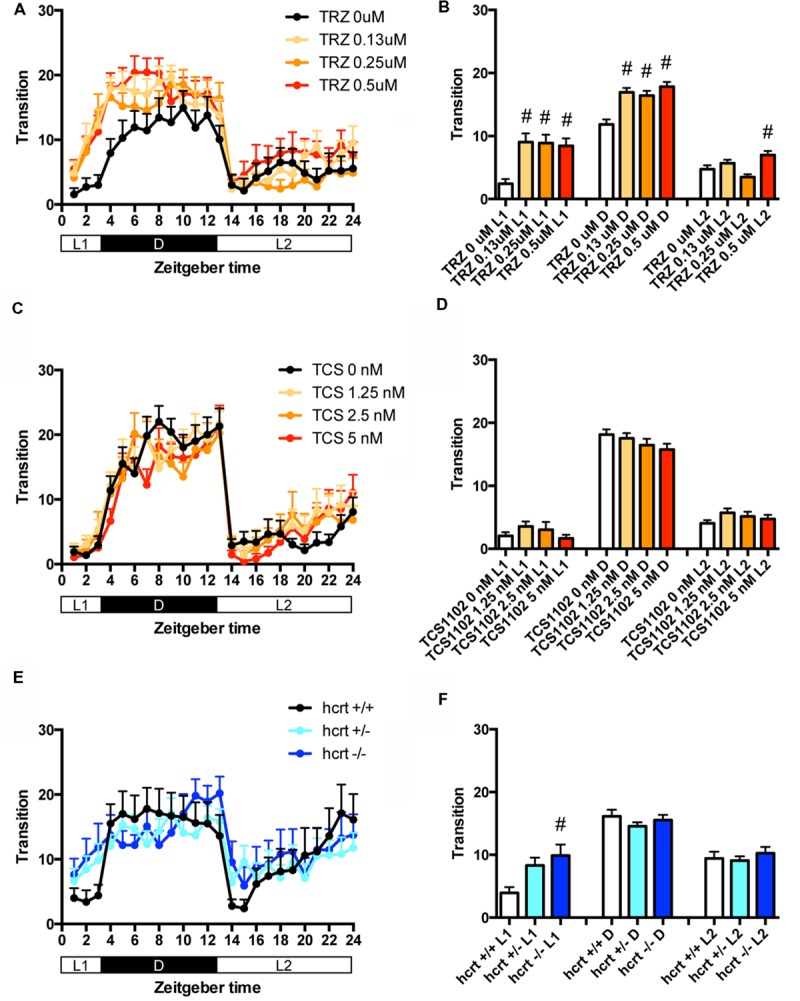
**Effects of triazolam, TCS1102, and hcrt knockout on the transition between rest and active states of zebrafish.** The number of transitions of zebrafish treated with triazolam **(A)**, TCS1102 **(C)**, and hcrt knockout **(E)** from 9 to 10 dpf are shown during the hour. The number of transitions of zebrafish treated with triazolam **(B)**, TCS1102 **(D)** and hcrt knockout **(F)** from 9 to 10 dpf are shown during L1, D, and L2. The data are represented as means with the standard error of mean. #*p* < 0.05 vs. control.

We then assessed the effect of TRZ, a common benzodiazepine hypnotic, TCS-1102, a dual antagonist of hcrt receptor (Bergman et al., 2008), and hcrt-KO on the transition between rest and active states of zebrafish. We generated hcrt-KO zebrafish using TALEN (Supplementary Figure S3). TRZ significantly increased the transition during L1 and D at all concentrations (**Figures 3A,B** and Supplementary Table S1). TCS-1102 did not cause significant effects on the transition (**Figures 3C,D** and Supplementary Table S1). hcrt-KO significantly increased the transition during L1 (**Figures 3E,F** and Supplementary Table S1).

**FIGURE 4:**
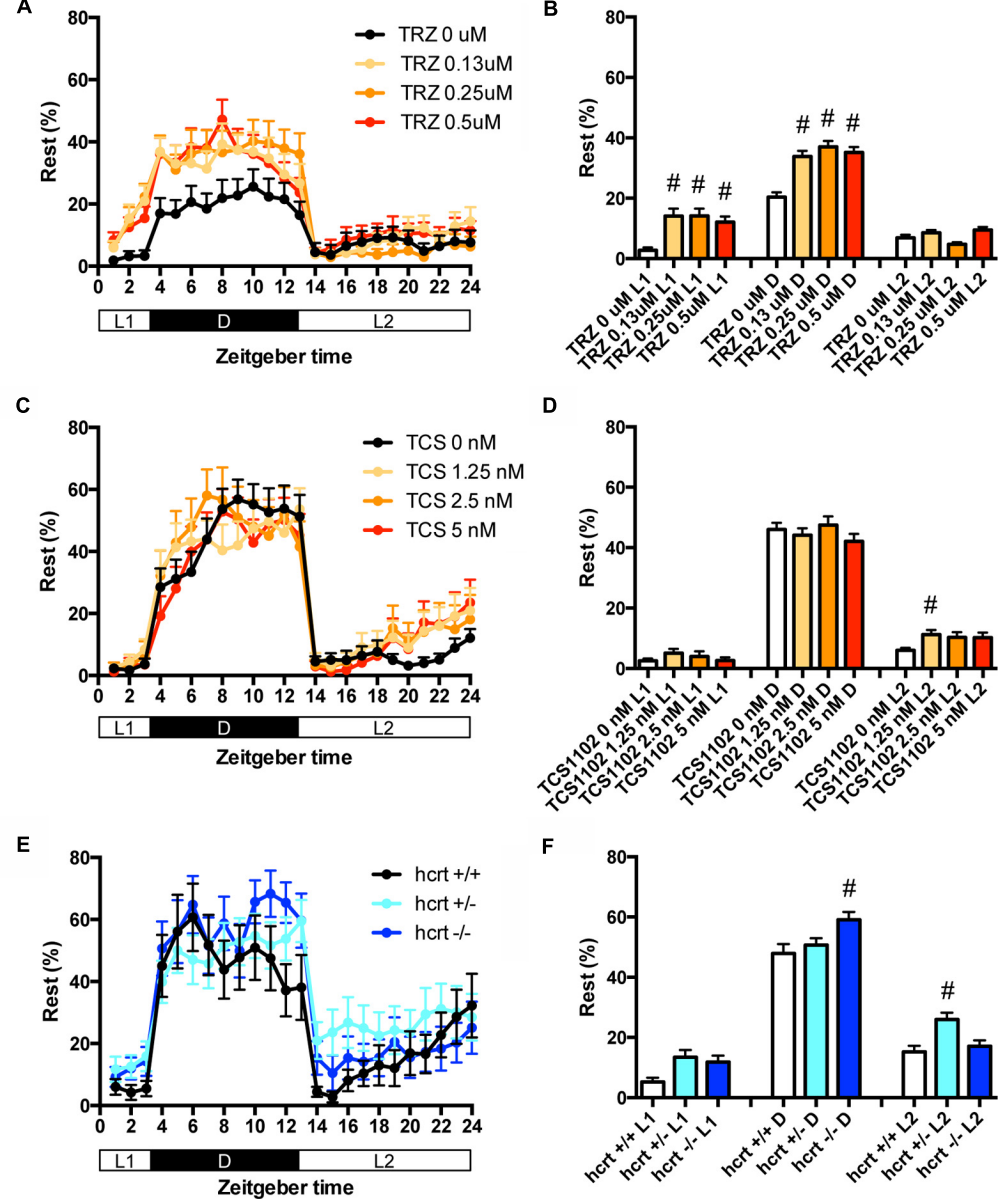
**Effects of triazolam, TCS1102, and hcrt knockout on the rest state of zebrafish.** The rest state of zebrafish treated with triazolam **(A)**, TCS1102 **(C)**, and hcrt knockout **(E)** from 9 to 10 dpf are shown as percentage of total behavior. The rest state of zebrafish treated with triazolam **(B)**, TCS1102 **(D)**, and hcrt knockout **(F)** from 9 to 10 dpf are shown as percentage in L1, D, and L2. The data are represented as means with the standard error of mean. #*p* < 0.05 vs. control.

We then assessed the effect of TRZ, TCS-1102, and hcrt-KO on the percentage in the rest state. TRZ significantly increased the percentage during L1 and D at all concentrations (**Figures 4A,B** and Supplementary Table S2). TCS-1102 significantly increased the percentage during L2 at 1.25 μM (**Figures 4C,D** and Supplementary Table S2). hcrt-KO significantly increased the percentage during D (**Figures 4E,F** and Supplementary Table S2).

These results suggest that TRZ may increase the percentage in the rest state by decreasing the duration of wake state in zebrafish (Supplementary Figure S2C, Pattern 2), which is similar to the effect of TRZ in mammals (Keighley et al., 1980). The results also suggest that TCS-1102 increase the percentage in the rest state in L2 by decreasing and increasing wake and rest state, respectively, in zebrafish (Supplementary Figure S2C, Pattern 3). The results also indicate that hcrt-KO may increase the percentage in the rest state in D by decreasing and increasing wake and sleep state, respectively (Supplementary Figure S2C, Pattern 3). hcrt-KO also increased the transition between sleep and rest states without affecting on the percentage in the rest state, suggesting that hcrt-KO may decrease the duration of rest and wake states in zebrafish (Supplementary Figure S2C, Pattern 4). These results suggest that both GABA-A modulators and blocking hcrt signaling may increase sleep state and that hcrt-KO may cause an increase in transition between sleep and awake states in zebrafish as observed in mammals (Baumann and Bassetti, 2005; Baier et al., 2011).

### Psychostimulants Decreased the Rest State of Zebrafish

**FIGURE 5:**
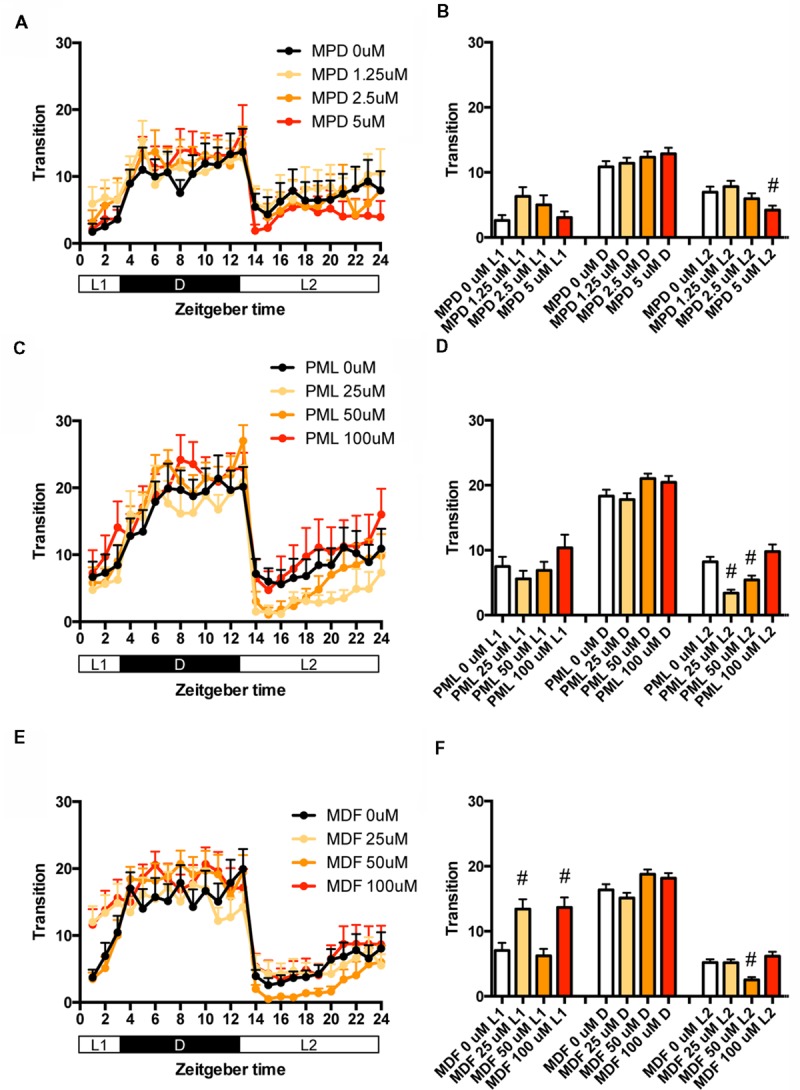
**Effects of methylphenidate, pemoline, and modafinil on the transition between rest and active states of zebrafish.** The number of transitions of zebrafish treated with methylphenidate **(A)**, pemoline **(C)**, and modafinil **(E)** from 9 to 10 dpf is indicated during the hour. The number of transitions of zebrafish treated with methylphenidate **(B)**, pemoline **(D)**, and modafinil **(F)** from 9 to 10 dpf is indicated as during L1, D, and L2. The data are represented as means with the standard error of mean. #*p* < 0.05 vs. control.

We then examined the effect of clinical psychostimulants on the transition between rest and active states of zebrafish. The transition in L2 was significantly decreased by MPD at 5 μM (**Figures 5A,B** and Supplementary Table S1), PML at 25 and 50 μM (**Figures 5C,D** and Supplementary Table S1) and MDF at 50 μM (**Figures 5E,F** and Supplementary Table S1). MDF at 25 and 100 μM also significantly increased the transition in L1 (**Figures 5E,F** and Supplementary Table S1).

**FIGURE 6:**
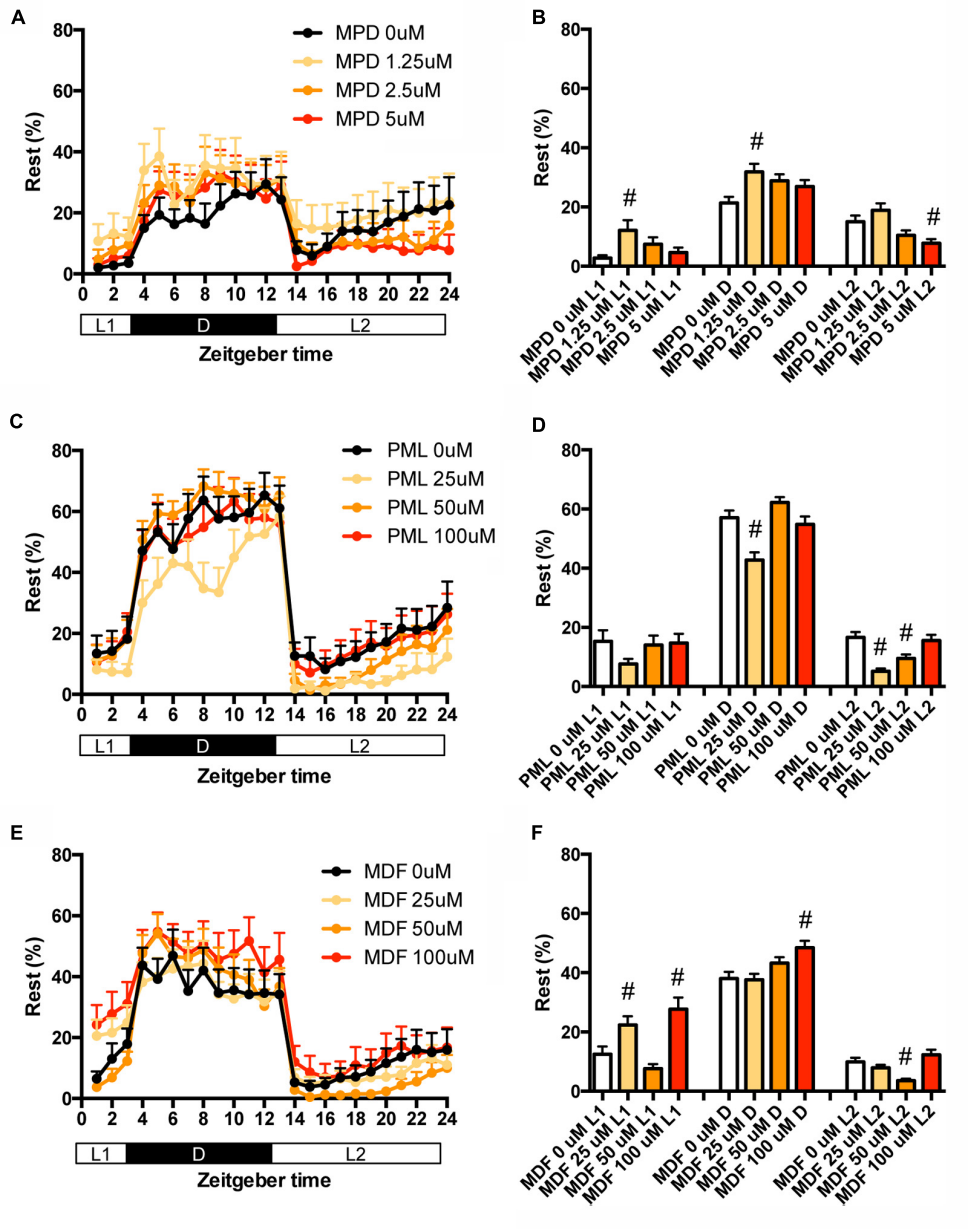
**Effects of methylphenidate, pemoline, and modafinil on the rest state of zebrafish.** The rest state of zebrafish treated with methylphenidate **(A)**, pemoline **(C)**, and modafinil **(E)** from 9 to 10 dpf is indicated as percentage of total behavior during the hour. The rest state of zebrafish treated with methylphenidate **(B)**, pemoline **(D)**, and modafinil **(F)** from 9 to 10 dpf is indicated as percentage in L1, D, and L2. The data are represented as means with the standard error of mean. #*p* < 0.05 vs. control.

We also examined the effect of clinical psychostimulants on the percentage in the rest state of zebrafish. The percentage in L2 was significantly decreased by MPD (**Figures 6A,B** and Supplementary Table S2), PML (**Figures 6C,D** and Supplementary Table S2) and MDF (**Figures 6E,F** and Supplementary Table S2). MDF also significantly increased the percentage in L1 (**Figures 6E,F** and Supplementary Table S2). These effects were the same as those in the transition between rest and active states. MPD at 2.5 μM in L1 and D (**Figures 6A,B** and Supplementary Table S2) and MDF at 100 μM in D also significantly increased the percentage, whereas PML at 25 μM significantly decreased the percentage in D (**Figures 6C,D** and Supplementary Table S2).

These results suggest that these psychostimulants may decrease the percentage in the rest state in L2 by increasing the duration of the wake state in zebrafish (Supplementary Figure S2C, Pattern 5), which is consistent with the effect of these psychostimulants in mammals (Nicholson and Pascoe, 1990; Edgar and Seidel, 1997; Lahti et al., 2009). The reasons why MPD and MDF increased the percentage in the rest states in L1 and D are currently unknown. Taken together, these results suggest that MPD, PML and MDF may decrease sleep state by increasing the awake state in zebrafish as observed in mammals (Banerjee et al., 2004; Sinita and Coghill, 2014).

### Behavioral Profiling of Zebrafish Treated with Hypnotics

It has been demonstrated that GABA-A receptor modulators can promote sleep with the impairment of locomotor performance, whereas hcrt receptor antagonists can promote sleep without locomotor impairment (Steiner et al., 2011; Ramirez et al., 2013). Therefore, we examined whether behavioral profiling could distinguish GABA-A receptor modulators and hcrt receptor antagonists.

We first classified zebrafish behavior into three groups based on their mobility: high, medium, and low. We then measured four behavioral endpoints, the Distance Moved (DM), Distance to the Zone in the center of the well (DZ), Frequency of entering the center Zone (FZ), and Turn Angle (TA) in the time showing each mobility, resulting in 12 measured endpoints (four behavioral endpoints at three mobility states).

**FIGURE 7:**
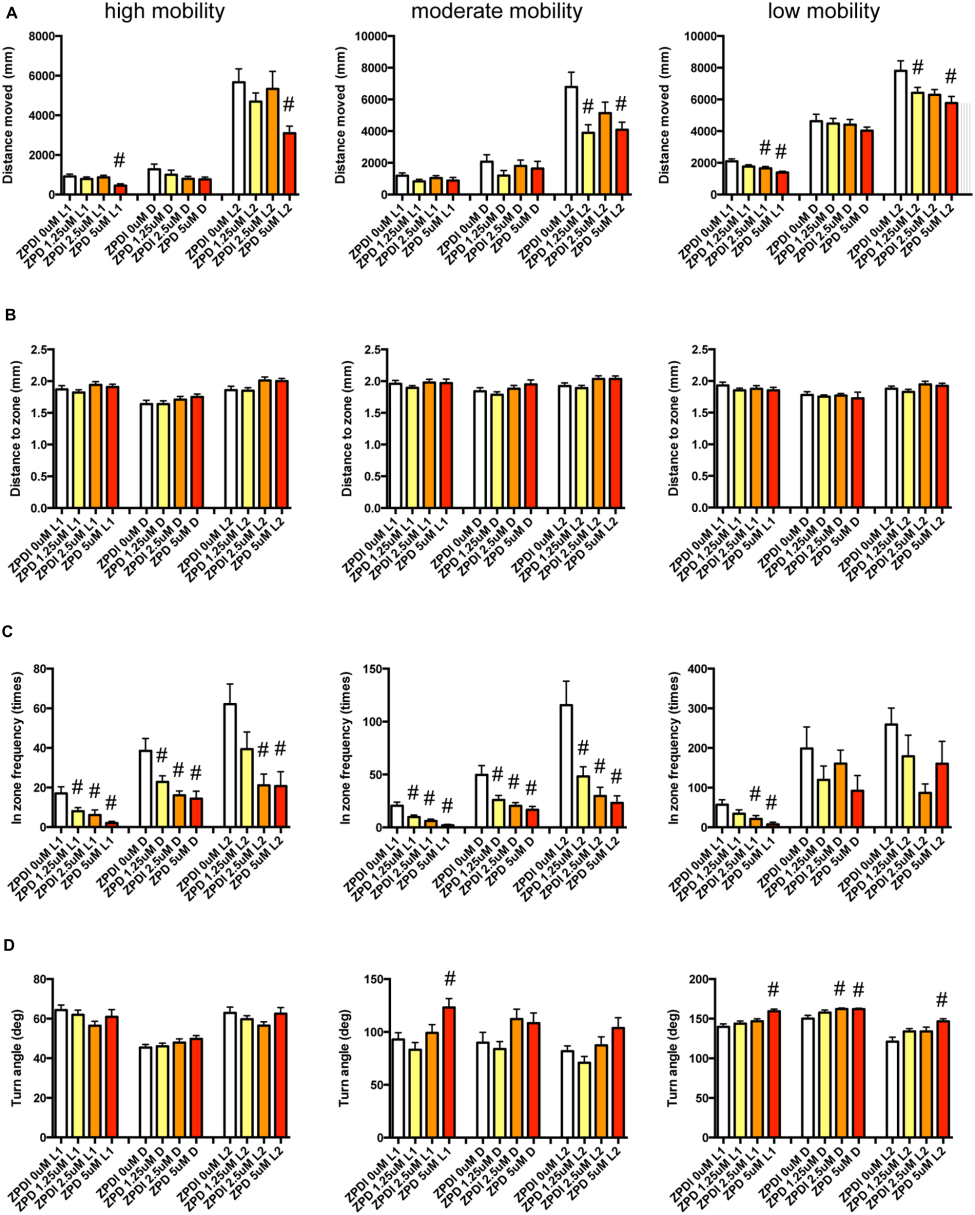
**Behavioral profiling of zebrafish treated with zolpidem.** The behavior of zebrafish treated with zolpidem from 9 to 10 dpf are classified into three groups based on their mobility. Distance moved **(A)**, distance to zone **(B)**, in-zone frequency **(C)**, and turn angle **(D)** at L1, D, and L2 periods are shown for each mobility classification. The data are represented as means with the standard error of mean. #*p* < 0.05 vs. 0 μM.

**FIGURE 8:**
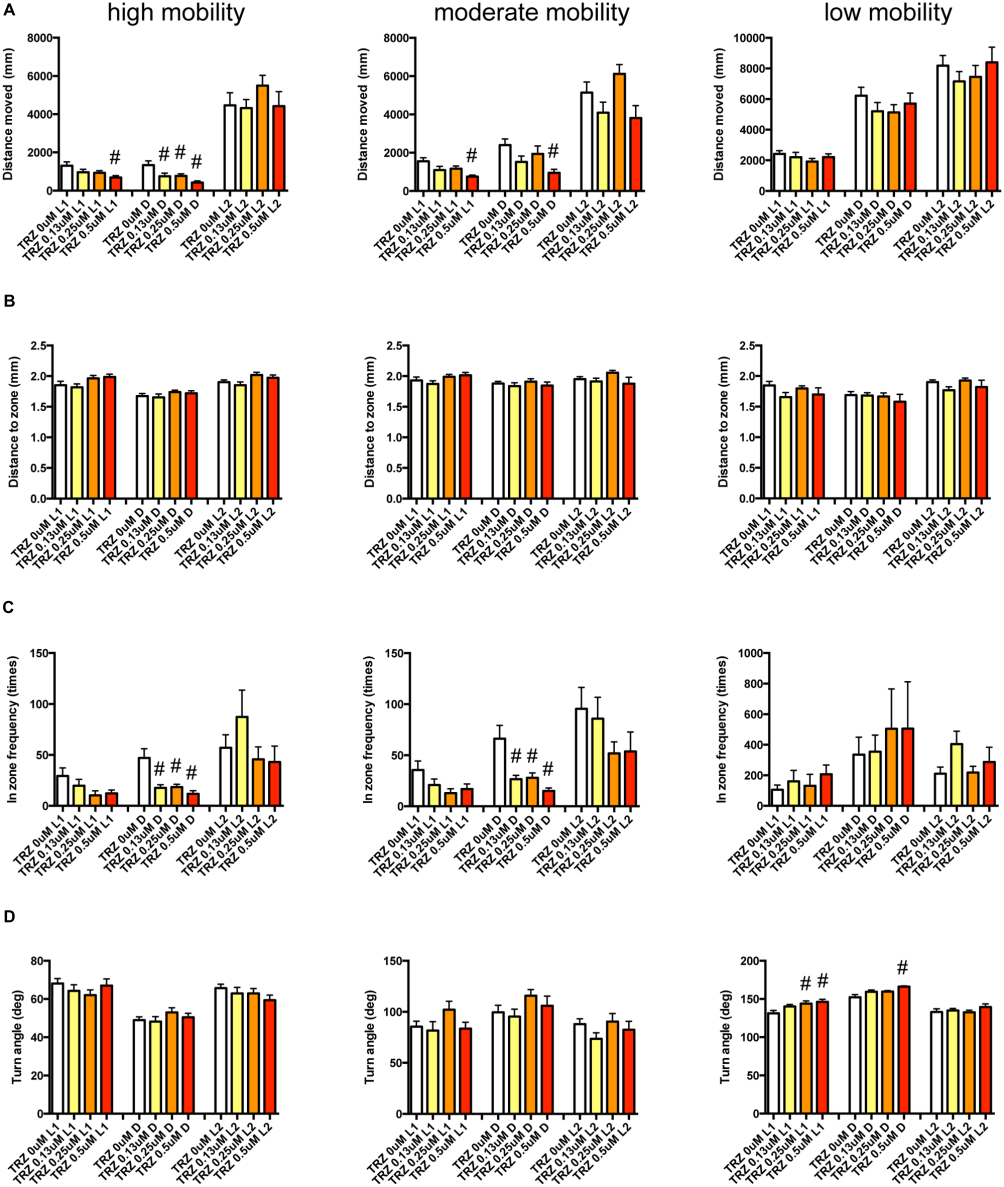
**Behavioral profiling of zebrafish treated with triazolam.** The behavior of zebrafish treated with triazolam from 9 to 10 dpf were classified into three groups based on their mobility. Distance moved **(A)**, distance to zone **(B)**, in zone frequency **(C)**, and turn angle **(D)** at L1, D, and L2 periods are shown for each mobility. The data were represented as means with the standard error of mean. #*p* < 0.05 vs. 0 μM.

**FIGURE 9:**
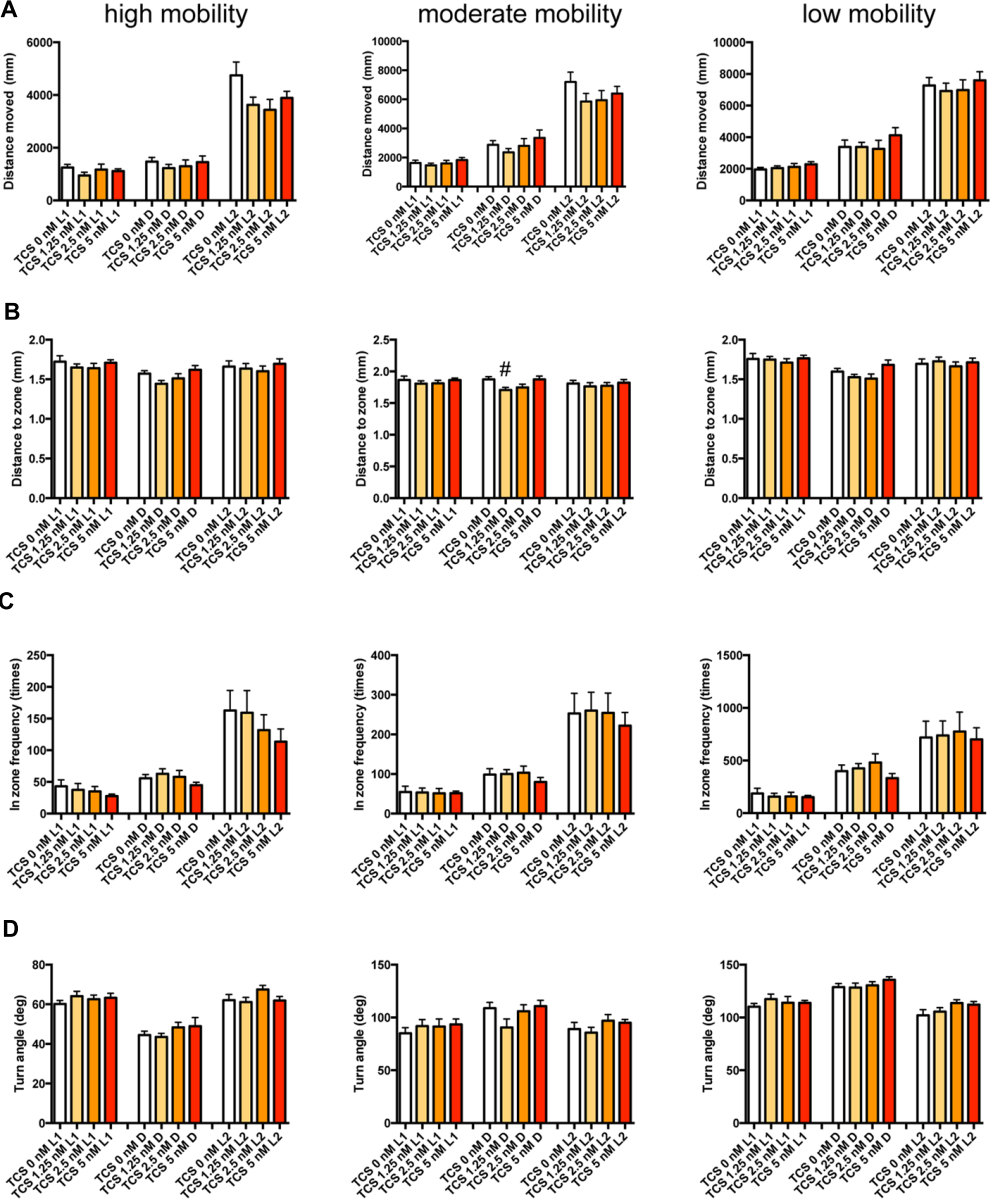
**Behavioral profiling of zebrafish treated with TCS-1102.** The behavior of zebrafish treated with TCS-1102 from 9 to 10 dpf was classified into three groups based on their mobility. Distance moved **(A)**, distance to zone **(B)**, in zone frequency **(C)**, and turn angle **(D)** at L1, D, and L2 periods are shown for each mobility classification. The data are represented as means with the standard error of mean. #*p* < 0.05 vs. 0 μM.

**FIGURE 10:**
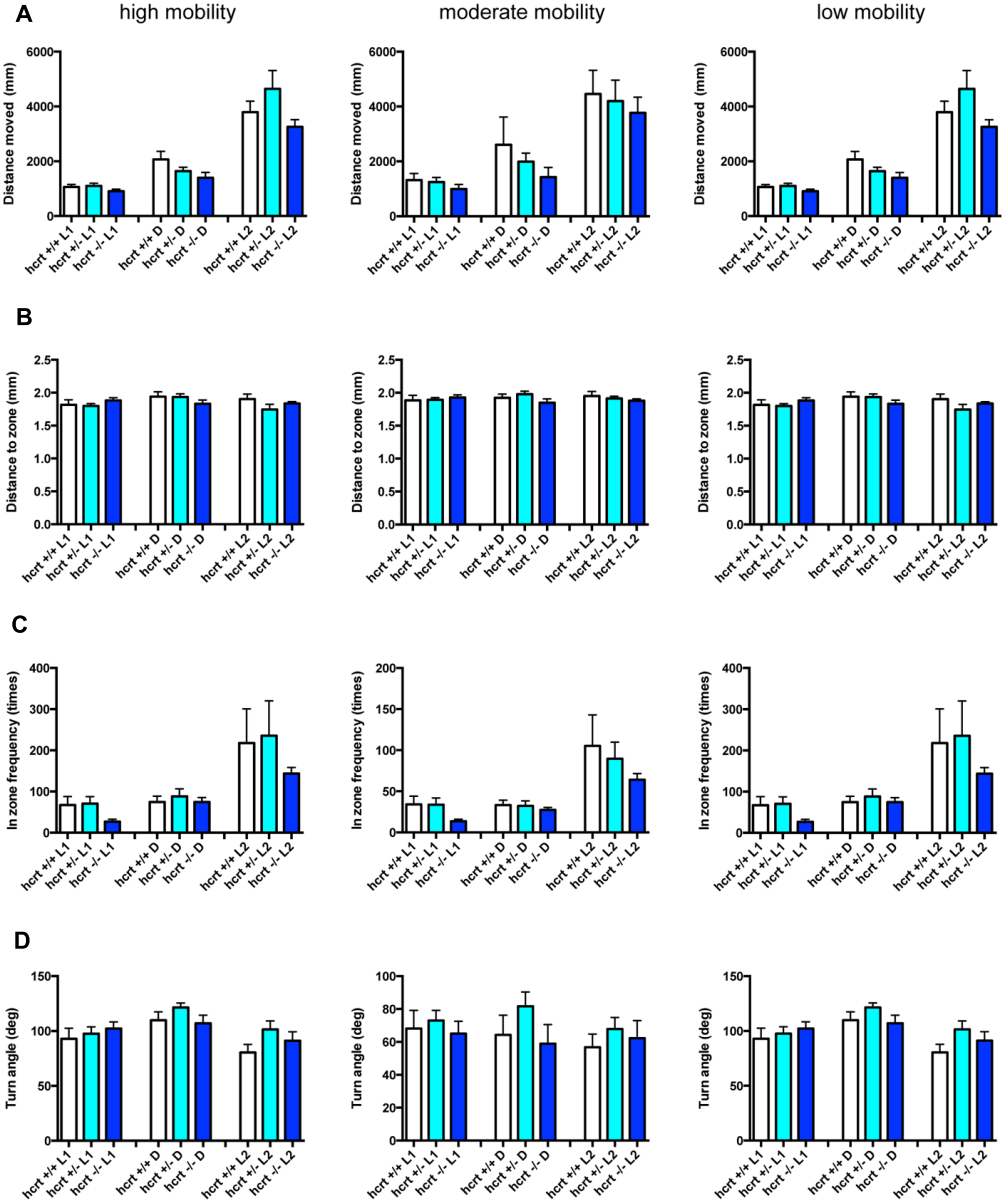
**Behavioral profiling of hcrt-KO zebrafish.** The behavior of hcrt-KO zebrafish from 9 to 10 dpf were classified into three groups based on their mobility. Distance moved **(A)**, distance to zone **(B)**, in zone frequency **(C)**, and turn angle **(D)** at L1, D, and L2 periods are shown for each mobility classification. The data are represented as means with the standard error of mean.

We examined the effect of ZPD (**Figure 7**, Supplementary Table S3-1), TRZ (**Figure 8**, Supplementary Table S3-2), TCS-1102 (**Figure 9**, Supplementary Table S3-3), and hcrt-KO (**Figure 10**, Supplementary Table S3-4) on the 12 endpoints.

As shown in **Figure 7** and Supplementary Table S3-1, ZPD significantly affected DM and FZ at high mobility and DM, FZ, and TA at medium and low mobility. DM in L1 and/or L2 were significantly decreased by ZPD at high, medium, and low mobilities. FZ was also significantly decreased by ZPD in L1, D, and L2 at high and medium mobility and L1 at low mobility. TA was significantly increased by ZPD in L1, D, and L2 at low mobility and L1 at medium mobility.

As shown in **Figure 8** and Supplementary Table S3-2, TRZ significantly affected DM and FZ at high and medium mobility and TA at low mobility. DM in L1 and D were significantly decreased by TRZ at high and medium mobility. FZ were also significantly decreased by TRZ in D at high and medium mobility. TA was significantly increased by TRZ in L1 and D at low mobility.

The decrease of DM in L1 at high mobility, the decrease of FZ in D at high and medium mobility, and the increase of TA in L1 and D at low mobility were common between the effects of ZPD and TRZ, suggesting that these behavioral endpoints may be related to the effects of hypnotics targeting GABA-A receptors.

As shown in **Figure 9** and Supplementary Table S3-3, TCS-1102 significantly affected DZ in D at medium mobility.

As shown in **Figure 10** and Supplementary Table S3-4, there were no significant changes in the 12 endpoints among hcrt+/+, hcrt+/–, and hcrt–/– zebrafish.

These results suggest that the GABA-A modulator may promote sleep with the impairment of locomotor performance, whereas blocking the hcrt signaling may promote sleep with little impairment of locomotor performance in zebrafish as observed in mammals (Steiner et al., 2011; Ramirez et al., 2013).

### Behavioral Profiling of Zebrafish Treated with Psychostimulants

**FIGURE 11:**
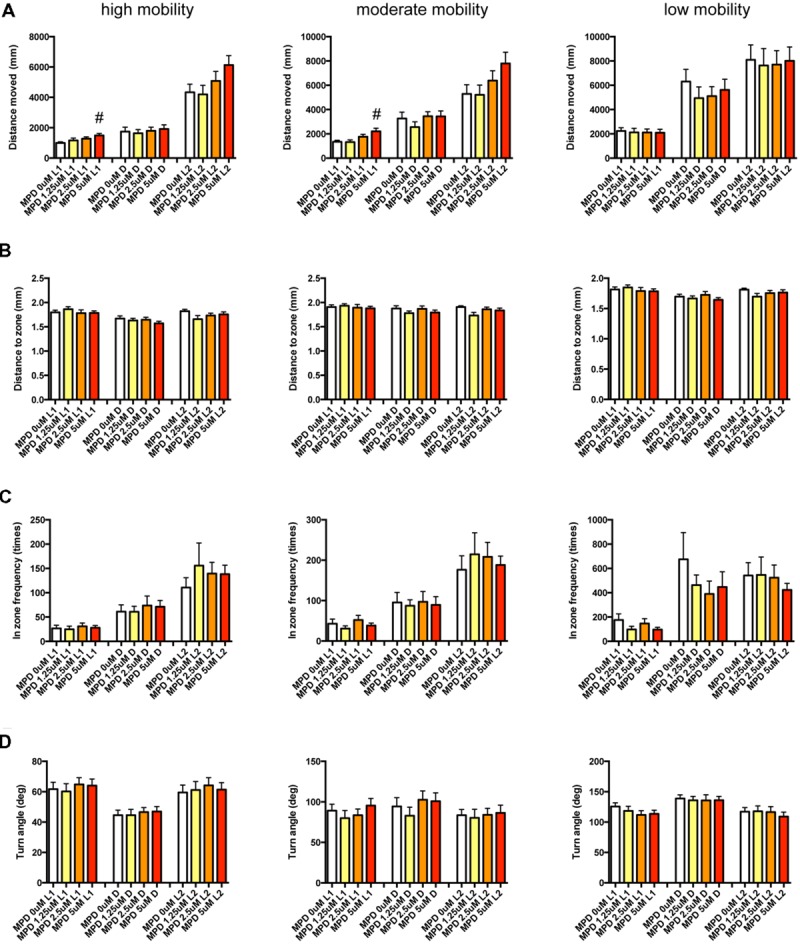
**Behavioral profiling of zebrafish treated with methylphenidate.** The behavior of zebrafish treated with methylphenidate from 9 to 10 dpf was classified into three groups based on mobility. Distance moved **(A)**, distance to zone **(B)**, in zone frequency **(C)**, and turn angle **(D)** at L1, D, and L2 periods are indicated for each mobility classification. The data are represented as means with the standard error of mean. #*p* < 0.05 vs. 0 μM.

**FIGURE 12:**
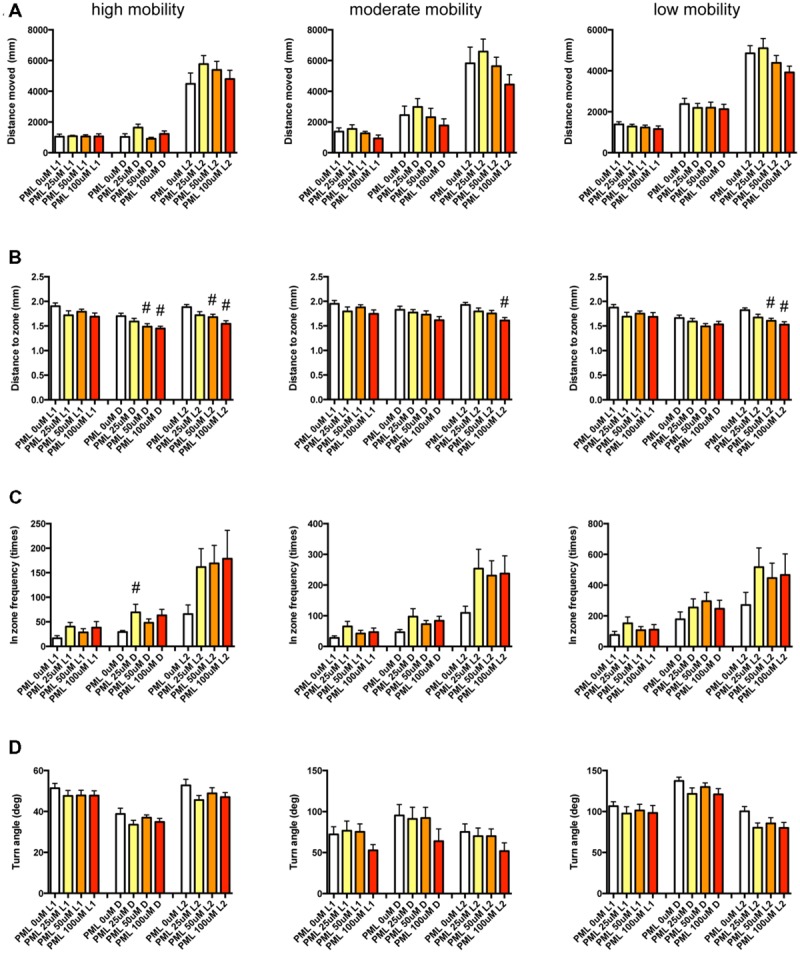
**Behavioral profiling of zebrafish treated with pemoline.** The behavior of zebrafish treated with pemoline from 9 to 10 dpf were classified into three groups based on their mobility. Distance moved **(A)**, distance to zone **(B)**, in zone frequency **(C)**, and turn angle **(D)** at L1, D, and L2 periods are shown for each mobility classification. The data are represented as means with the standard error of mean. #*p* < 0.05 vs. 0 μM.

**FIGURE 13:**
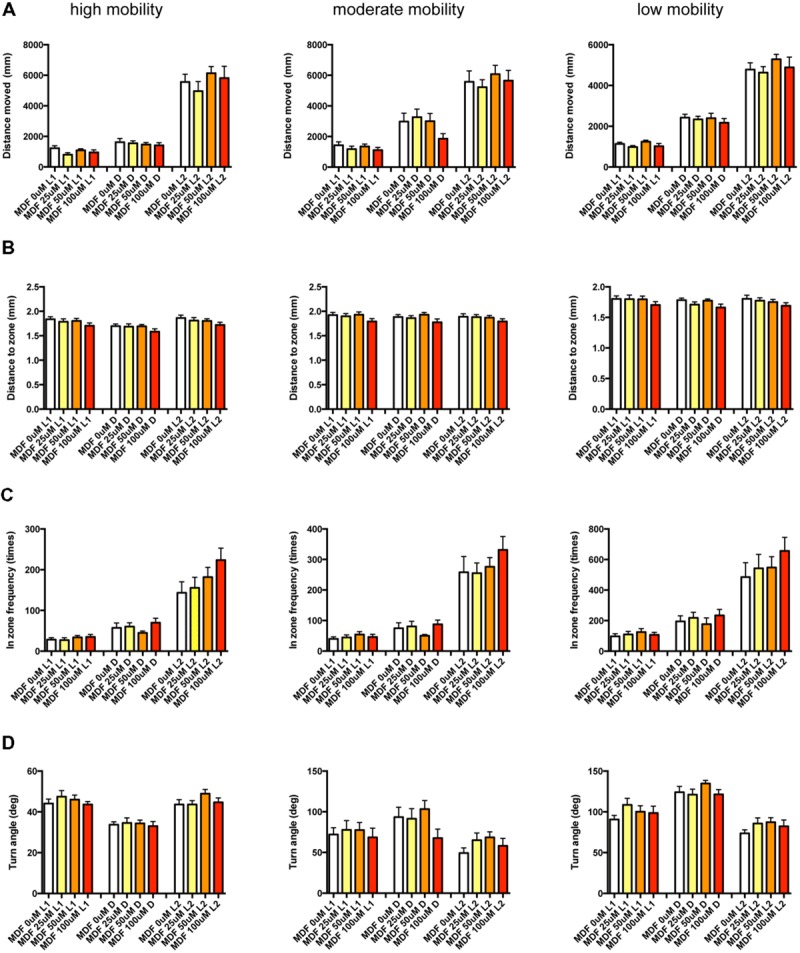
**Behavioral profiling of zebrafish treated with modafinil.** The behavior of zebrafish treated with modafinil from 9 to 10 dpf was classified into three groups based on mobility. Distance moved **(A)**, distance to zone **(B)**, in zone frequency **(C)**, and turn angle **(D)** at L1, D, and L2 periods are shown for each mobility classification.

It has been demonstrated that sympathomimetic psychostimulants can increase wakefulness causing autonomic arousal and psychomotor agitation, whereas non-sympathomimetic psychostimulants can promote wakefulness in the absence of the other arousing effects typically seen with the sympathomimetic psychostimulants (Banerjee et al., 2004). MPD and PML are representative sympathomimetic psychostimulants, whereas the pharmacodynamics of MDF as a psychostimulant has not been fully elucidated. Therefore, we examined the effect of MPD (**Figure 11**, Supplementary Table S3-5), PML (**Figure 12**, Supplementary Table S3-6), and MDF (**Figure 13**, Supplementary Table S3-7) on the 12 endpoints.

As shown in **Figure 11**, Supplementary Table S3-5, DM in L1 at high and medium mobility was significantly increased by MPD.

As shown in **Figure 12** and Supplementary Table S3-6, DZ was significantly decreased by PML. At high mobility, DZ was significantly decreased by PML in D and L2. At medium and low mobility, DZ was significantly decreased by PML in L2.

As shown in **Figure 13** and Supplementary Table S3-7, there was no significant change in the 12 endpoints induced by MDF.

These results suggest that sympathomimetic psychostimulants such as MPD and PML may increase wakefulness with changing locomotor performance, whereas MDF may increase wakefulness without impairments of locomotor performance in zebrafish as observed in mammals (Banerjee et al., 2004)

### Clustering of Zebrafish Behavior Distinguished Sleep-wake Modifiers based on their Mode of Action

**FIGURE 14:**
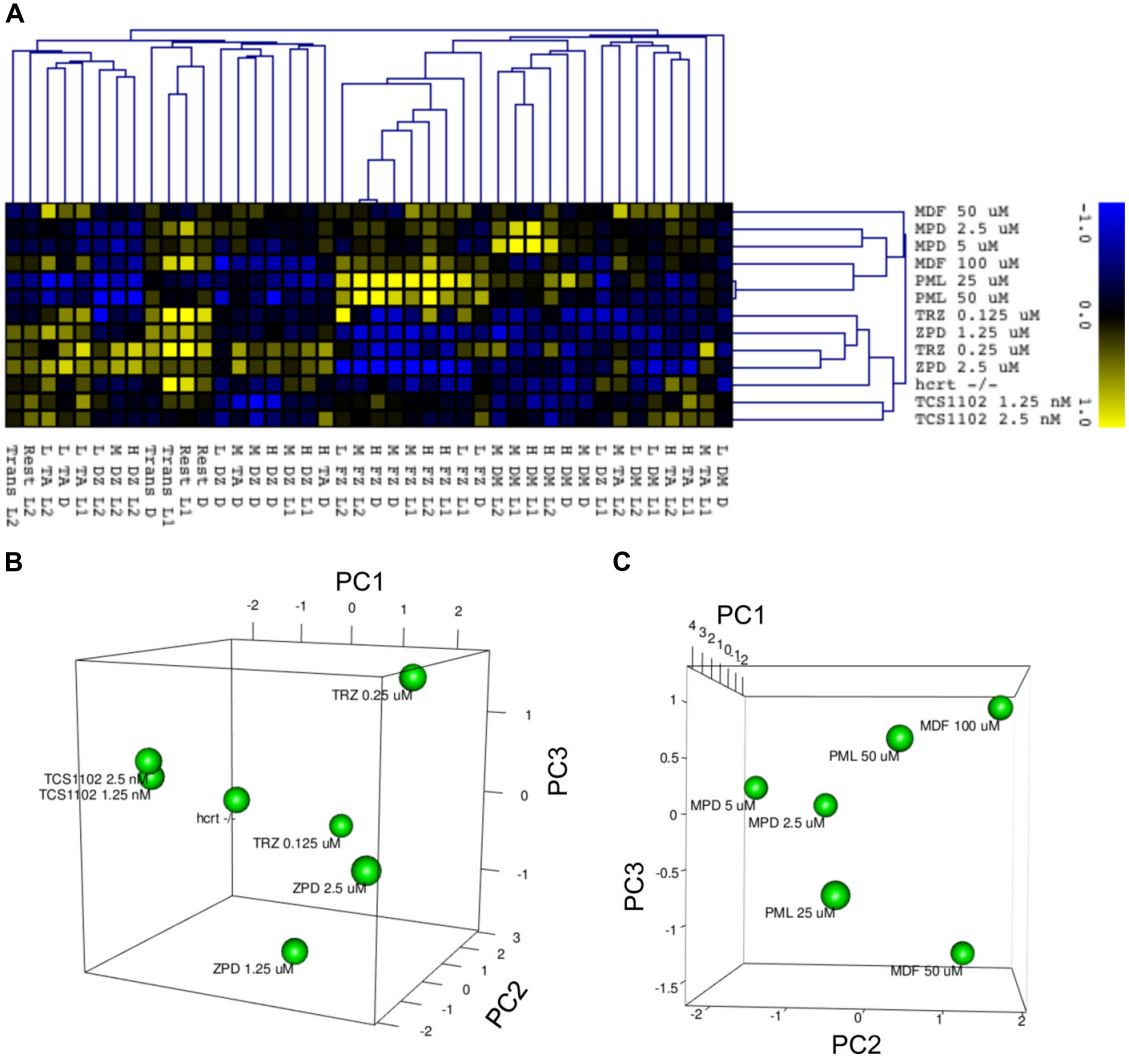
**HCL and PCA of behavior of zebrafish treated with sleep/awake modifiers. (A)** HCL of behavior of zebrafish treated with sleep/awake modifiers. Z scores of each behavioral endpoint were subjected to HCL using Spearman correlation with average linkage. **(B)** PCA of behavior of zebrafish treated with hypnotics and hcrt-KO. Z scores of each behavioral endpoint were subjected to PCA. **(C)** PCA of behavior of zebrafish treated with psychostimulants. Z scores of each behavioral endpoint were subjected to PCA.

We finally examined whether behavioral profiling in zebrafish treated with sleep-wake modifiers could be used to distinguish the modes of action of different modifiers. Using the quantitative data matrix from 42 endpoints (14 behavioral endpoints in L1, D, and L2) analyzed for the sleep-wake modifiers, we performed HCL analysis, which has been used in transcriptome analysis to classify the samples based on the expression profile (Nishimura et al., 2007). As shown in **Figure 14A**, the HCL of the Z score of each behavioral endpoint clearly distinguished hypnotics and psychostimulants. Moreover, the HCL was able to classify sleep modifiers targeting GABA-A signaling (ZPD and TRZ) and hcrt signaling (TCS-1102 and hcrt-KO).

We also performed PCA to examine whether the behavioral profiling could distinguish hypnotics based on their mode of action. PCA has also been widely used in transcriptome analysis to classify the samples, but it uses a different algorithm than HCL (Nishimura et al., 2007). As shown in **Figure 14B**, PCA of the Z score in our analysis using behavioral profiling of zebrafish treated with hypnotics revealed that principal component (PC) 1 mainly differentiated GABA-A modifiers from the hcrt receptor antagonist/hcrt-KO. FZ at high mobility in D negatively contributed to the PC1 (Supplementary Table S4-1). In D, FZ at high mobility negatively correlated with TA at low mobility (*r* = -0.923) and positively correlated to FZ at medium mobility (*r* = 0.977; Supplementary Table S5). These endpoints were significantly affected by ZPD (**Figure 7** and Supplementary Table S3-1) and TRZ (**Figure 8** and Supplementary Table S3-2). These results suggest that FZ at high mobility in D may be a key behavioral endpoint for distinguishing the GABA-A modulator and the hcrt receptor antagonist.

We also examined whether PCA of behavioral profiling could distinguish psychostimulants based on their mode of action. As shown in **Figure 14C**, PCA of the Z score of behavioral profiling of zebrafish treated with psychostimulants revealed that PC2 mainly distinguished representative sympathomimetic psychostimulants (e.g., MDF and PML) and MDF. DM at high mobility in D negatively contributed to PC2 (Supplementary Table S4-2). DM at high mobility in D was significantly affected by PML although multiple comparisons between the controls revealed no significant change by PML (**Figure 12** and Supplementary Table S3-6). DM at high mobility in D was positively correlated to DM at high mobility in L1 (*r* = 0.550; Supplementary Table S5), which was significantly increased by MPD (**Figure 11** and Supplementary Table S3-5). These results suggest that DM at high mobility in L1 and D may be key behavioral endpoints for distinguishing representative sympathomimetic psychostimulants and MDF.

## Discussion

In this study, we demonstrated that behavioral profiling of zebrafish treated with clinically used hypnotics or psychostimulants between 9 and 10 dpf can be used to classify these drugs based on their mode of action. We also identified behavioral endpoints distinguishing sleep-wake modifiers based on their mode of action.

### Behavioral Profiling of Zebrafish between 9 and 10 dpf can be Used to Classify Sleep-wake Modifiers

It has been demonstrated that behavioral profiling of zebrafish can be used to classify neuroactive compounds based on their mode of action (Rihel and Schier, 2013; Stewart et al., 2015). Kokel et al. (2010)performed a high-throughput screen of 14,000 compounds using photomotor response of zebrafish at 2 dpf and demonstrated that profiling the photometer response classified these compounds based on their mechanisms of action. Rihel et al. (2010) used an automated rest/wake behavioral assay to monitor the activity of zebrafish treated with small molecules from 4 to 7 dpf and demonstrated that behavioral profiling can reveal conserved functions of psychotropic molecules and predicted the mechanisms of action of poorly characterized compounds. However, to our knowledge, there have been no studies demonstrating the effects of clinically used benzodiazepines (i.e., TRZ, estazolam, quazepam, flurazepam, temazepam), Z-drugs (i.e., ZPD, eszopiclone, and zaleplon), and sympathomimetic psychostimulants (e.g., MPD and PML) on sleep-wake states of zebrafish larva.

In this study, we demonstrated that the hypnotic effects of ZPD at 9 dpf was stronger than that at 7 dpf. It has been demonstrated that the sensitivity to ZPD was increased during development in rats (Wall, 2005; Chudomel et al., 2009). The action of benzodiazepine site agonists depends on the subunit composition of GABA-A receptors (Wall, 2005; Chudomel et al., 2009). ZPD has selectivity for GABA-A receptors of the form α1βxγ2 (where x is 1, 2, or 3; Rudolph and Mohler, 2004). In rats, although the α1 subunit is present at birth, the expression of α1 is increased between postnatal days 14 and 21 (Poulter et al., 1992), which corresponds to around 10 dpf in zebrafish (Jomaa et al., 2014). Expression of α6 subunits can modify ZPD action by changing the proportion of α1βxγ2, α6β2/3γ2, and α1α6β2/3γ2 (Minier and Sigel, 2004). The increased sensitivity of ZPD from 9 to 10 dpf may be related to the expression level of α1 and α6 subunits. A study of spatial and temporal expression patterns of α1 and α6 subunits in the zebrafish brain during the development remains to be performed.

Using zebrafish larva at a relatively late stage for chemical screening has several advantages. The neuronal system, including the neural circuits and blood–brain barrier, is more mature than in the early stage so it is likely to increase the sensitivity and selectivity to chemicals targeting the central nervous system (Watanabe et al., 2012; Nishimura et al., 2013, 2015). The repertory of behavior is also increased during development (Kalueff et al., 2013; Nishimura et al., 2015; Stewart et al., 2015). However, using zebrafish larva at a relatively late stage decreases the throughput of chemical screening. The developmental stage used in chemical screens involving zebrafish should be chosen depending on the application (Rennekamp and Peterson, 2015).

### Behavioral Endpoints Distinguishing Hypnotics based on their Mode of Actions

In this study, we demonstrated that GABA-A receptor modulation was different following treatment with an hcrt receptor antagonist vs. hcrt-KO by FZ at high mobility during night conditions. This was negatively correlated to TA at low mobility and positively correlated to FZ at medium mobility.

Mobility is calculated by taking every pixel identified as the subject and comparing the value between the current image and the previous one. Therefore, mobility may be correlated to distance per movement for each endpoint related to wakefulness (Tejada et al., 2011). In rats, waking states can be divided into three different states, namely an awake exploratory behavior state when rats are actively moving, a grooming state when rats show grooming behavior, and an awake resting state when rats are immobile (Manabe et al., 2011). These findings motivated us to classify zebrafish behavior into three (high, medium, and low) mobility states and to analyze behavioral endpoints at each mobility state. Electroencephalography (Afrikanova et al., 2013) and/or optogenetics (Rihel and Schier, 2013) may reveal the exact relationship between these mobility states and wakefulness in zebrafish.

Behavioral profiling revealed that FZ at high mobility during the night was significantly decreased in zebrafish treated with ZPD and TRZ but not treated with TCS-1102 and hcrt-KO. FZ at high mobility during the night was positively correlated to DM at high mobility during night conditions. These results suggest that the decrease of FZ at high mobility during the night conditions may be related to the impairment of locomotion induced by ZPD and TRZ. These results are consistent with previous reports showing that the GABA-A receptor modulator can promote sleep with impairment of locomotor performance, whereas hcrt receptor antagonists and hcrt-KO can promote sleep without locomotor impairment (Kaur et al., 2008; Steiner et al., 2011; Elbaz et al., 2012; Ramirez et al., 2013).

The behavioral profiling also revealed that TA at low mobility during the night was significantly increased in zebrafish treated with ZPD and TRZ but not treated with TCS-1102 and hcrt-KO. In rodents, TA in the peripheral zone indicates non-locomotor movements of the body (Ruan et al., 2015), suggesting that both ZPD and TRZ may increase non-locomotor movement of the body at low mobility during the night. It has been demonstrated that ZPD produced muscle twitching and spasticity during hypnosis in GABA-A receptor α1 –/– mice (Kralic et al., 2002). The mechanism accounting for the increase of TA remains to be elucidated.

Zolpidem had greater effects than TRZ in the transition between rest and active states and the percentage in the rest state. The concentrations of ZPD used in this study were 10 times higher than those of TRZ. The EC50 of ZPD and TRZ on the α1β2γ2 GABA-A receptor were 78 and 52 nM, respectively (Sanna et al., 2002). The greater effect of ZPD on the transition between rest and active states and the percentage in the rest state over those in TRZ suggest that the α1β2γ2 GABA-A receptor may be involved in these endpoints.

The hcrt-KO zebrafish showed the increase of the transition between rest and active states and the percentage in the rest state. It has been well known that blocking hcrt signaling can cause the increase of state transition and the decrease of wake duration in mammals (Baumann and Bassetti, 2005; Baier et al., 2011). Based on these findings, we think that these behavioral changes might show symptoms of narcolepsy. It has also been demonstrated that exposure to light during the night increased locomotor activity in hcrt neuron-ablated zebrafish in contrast to the decrease in wild-type siblings (Elbaz et al., 2012). Sound stimulus during day also caused hypersensitive response in hcrt neuron-ablated zebrafish in contrast to the reduction of locomotor activity in wild-type siblings (Elbaz et al., 2012). We currently examine whether the hcrt-KO zebrafish we developed in this study can show these behaviors.

### Behavioral Endpoints Distinguishing Psychostimulants

In this study, we demonstrated that representative sympathomimetic psychostimulants MPD and PML were differentiated from MDF by DM at high mobility during L1 and D. In children, placebo-controlled studies have demonstrated that MPD increased motor activity at sleep onset (Ironside et al., 2010). In contrast, MDF increased wakefulness at the expense of slow-wave and paradoxical sleep with no increase in locomotor activity in hamsters (Webb et al., 2006). These reports may be consistent with our results demonstrating the significant increase of DM at high mobility in L1 by MPD and the significant increases of the number of transition between rest and active and the percentage in the rest state during L1 without significant effects on DM by MDF. These results suggest that DM at high mobility may be useful to distinguish representative sympathomimetic psychostimulants from other psychostimulants.

We demonstrated that the number of transition between rest and active states and the percentage in the rest state of zebrafish treated with MPD, PML, or MDF were significantly decreased in L2. However, the concentrations of MDF showing the decrease were very narrow. It has been demonstrated that sleep-promoting ventrolateral preoptic (VLPO) neurons are inhibited by dopamine via activation of α2 noradrenergic receptors and the noradrenergic inhibition of VLPO neurons is potentiated by MDF (Cornil et al., 2002; Gallopin et al., 2004; Vetrivelan et al., 2014). Although mammals have three subtypes of α2 noradrenergic receptors (α2A, α2B and α2C), zebrafish have five distinct α2 noradrenergic receptors (α2A, α2B, α2C, α2Da, and α2Db), which may make the zebrafish a2-adrenergic system more complicated (Ruuskanen et al., 2005) and may account for why the MDF effect is not dose-dependent.

### Advantages and Disadvantages of Behavioral Profiling in Zebrafish

Zebrafish can absorb a wide range of chemicals from the medium in which they swim. Zebrafish also exhibit a number of simple and complex neurobehavior which appear to be comparable at a functional level with human behavior. Because zebrafish are prolific and small, various neurobehaviors of larval zebrafish can be tracked in multi-well plates using automated and commercially available video-tracking systems. These tracking systems make it possible to undertake a systematic and objective analysis of zebrafish behaviors and to yield the statistical power necessary to detect subtle but significant changes in the behaviors in response to pharmacological and genetic interventions (Nishimura et al., 2015).

It has been demonstrated that zebrafish possessed orthologs for 86% of 1318 human drug targets (Gunnarsson et al., 2008). However, there can be interspecies differences in the pharmacodynamics. For example, dopamine D1 receptor agonists have opposite efficacies in zebrafish and mammals (Rihel et al., 2010). In this study, we demonstrated that TCS-1102 increased the percentage in the rest state. However, the effect was only observed in L2 at 1.25 nM. In contrast to mammals that have two hcrt receptors (Hcrtr1 and Hcrtr2), zebrafish has only one hcrt receptor with 60 and 70% identity to mammalian Hcrtr1 and Hcrtr2, respectively (Prober et al., 2006). The difference in the amino acid sequence of the zebrafish hcrt receptor may account for the relatively low sensitivity to the hcrt receptor antagonist in this study.

Despite these disadvantages, phenotype-based screening using zebrafish have successfully discovered novel therapeutic drugs and identified novel uses for existing drugs (MacRae and Peterson, 2015) and used for screening side effects of clinical drugs (Kanungo et al., 2014). Therefore, the profiling of zebrafish neurobehavior may be one of the screening systems that can detect both therapeutic and side effects of drugs with high sensitivity. Integration of the screening using zebrafish and the detailed characterization using rodents may minimize risks in the animal-human extrapolation (Sogorb et al., 2014).

In summary, we demonstrated that behavioral profiling of zebrafish from 9 to 10 dpf could classify sleep-wake modifiers based on their mode of action. Because zebrafish are highly amenable to genome editing and chemical screening, behavioral profiling can be useful in identifying genes related to sleep-wake disturbance associated with various diseases and novel therapeutic compounds for insomnia and excessive daytime sleep with low toxicity profiles.

## Author Contributions

YN conceived the study, analyzed the data, and wrote the manuscript. SO developed novel analytic tools using R. RK generated hcrt-KO zebrafish. SS, SM, YA, KK and RK performed experiments. TT conceived the study and wrote the manuscript.

## Conflict of Interest Statement

The authors declare that the research was conducted in the absence of any commercial or financial relationships that could be construed as a potential conflict of interest.

## Ethics Statement

Mie University Institutional Animal Care and Use Committee guidelines state that no approval is required for experiments using zebrafish. However, animal experiments described in this manuscript conform to the ethical guidelines established by the Institutional Animal Care and Use Committee at Mie University.

## Abbreviations

AD: Alzheimer’s disease
dpf: day per fertilization
fps: frame per second
HCL: hierarchical clustering
hcrt: hypocretin
MDF: modafinil
MPD: methylphenidate
mpf: month per fertilization
PCA: principal component analysis
PML: pemoline
TALEN: transcription activator-like effector nucleases
TRZ: triazolam
ZPD: zolpidem
