# Supplementary figures and images for: Pharmacological profiling of zebrafish behavior using chemical and genetic classification of sleep-wake modifiers
Source: Front Pharmacol. 2015 Nov 3;6:257. doi: 10.3389/fphar.2015.00257 (PMC4630575; doi:10.3389/fphar.2015.00257)

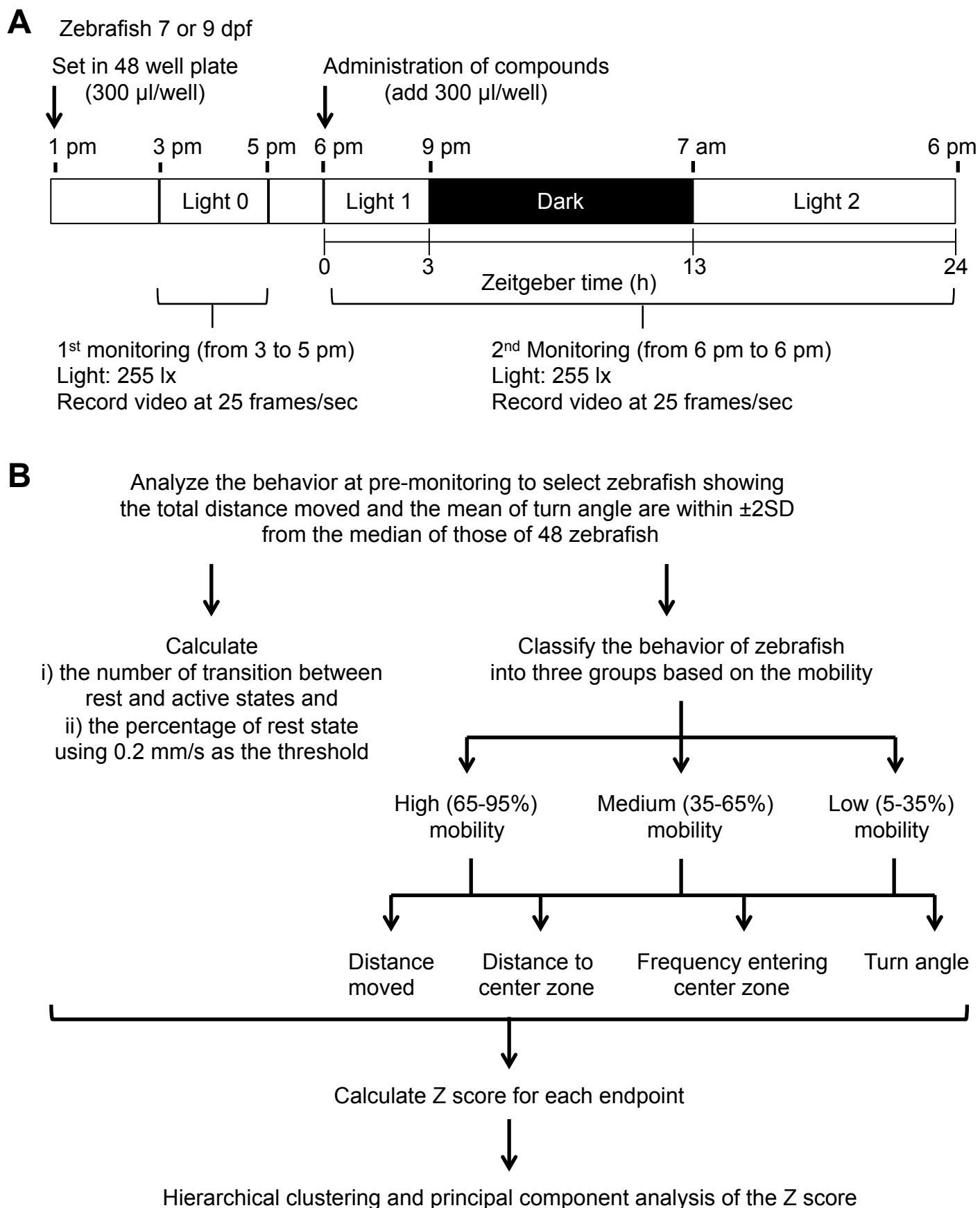

**Figure S1 Overview of the behavior analysis used in this study**

Supplement: Supplementary file 6 [file Image_1.PDF]

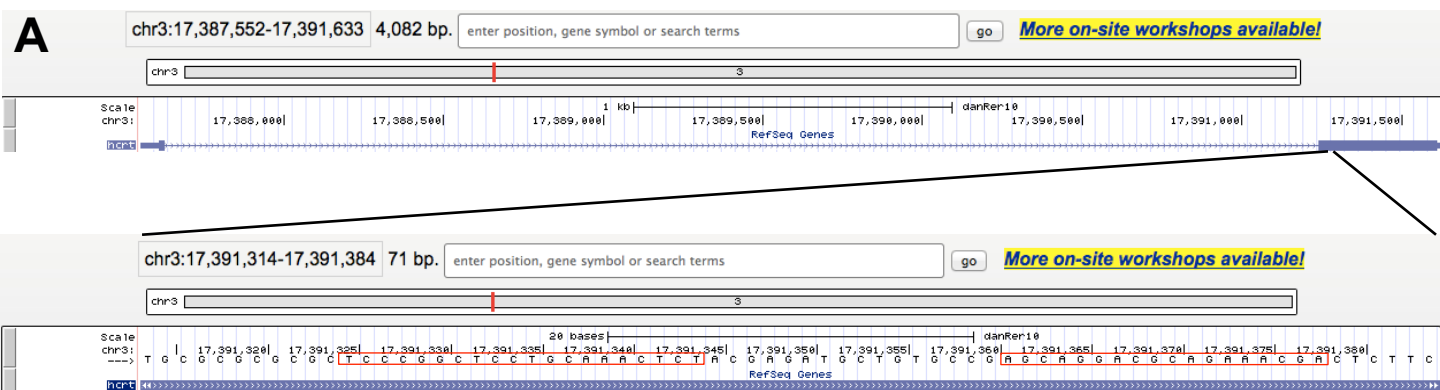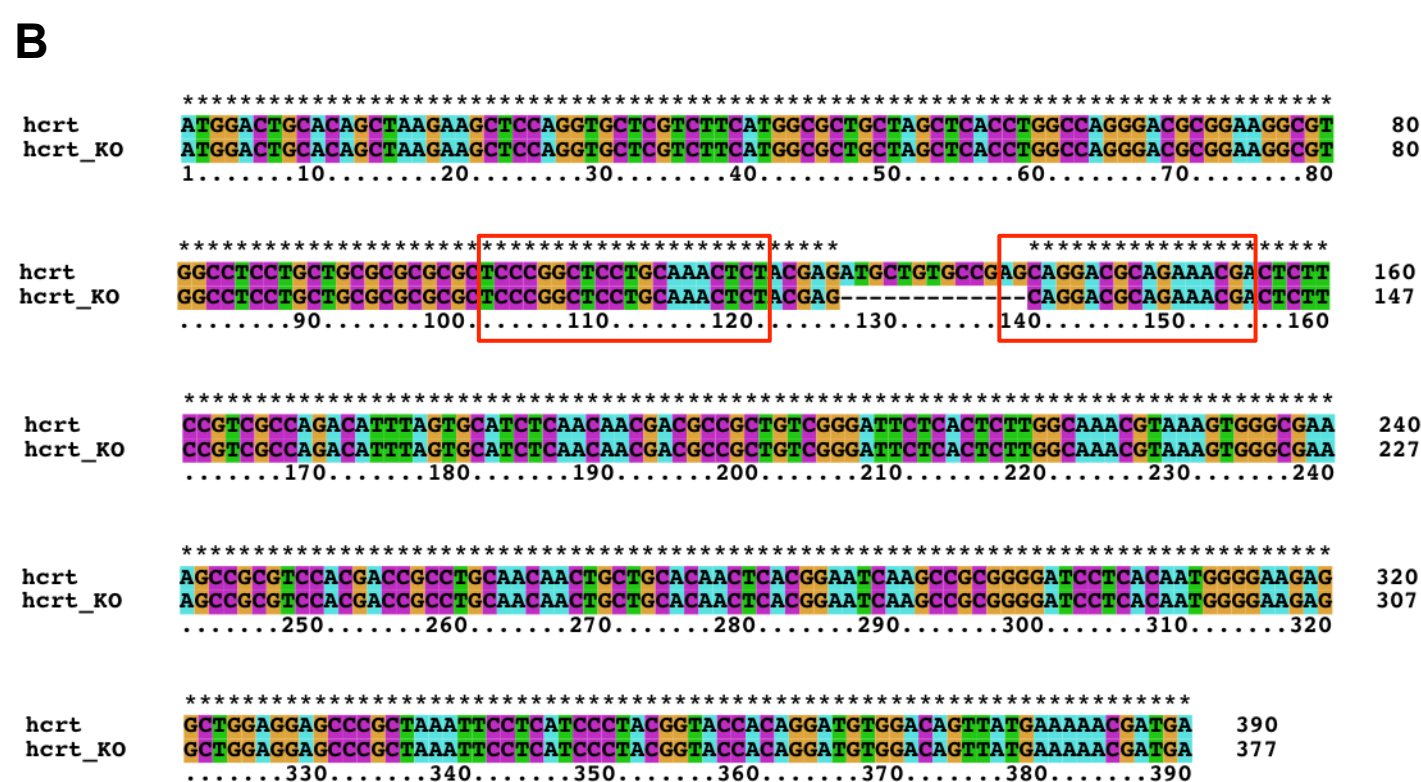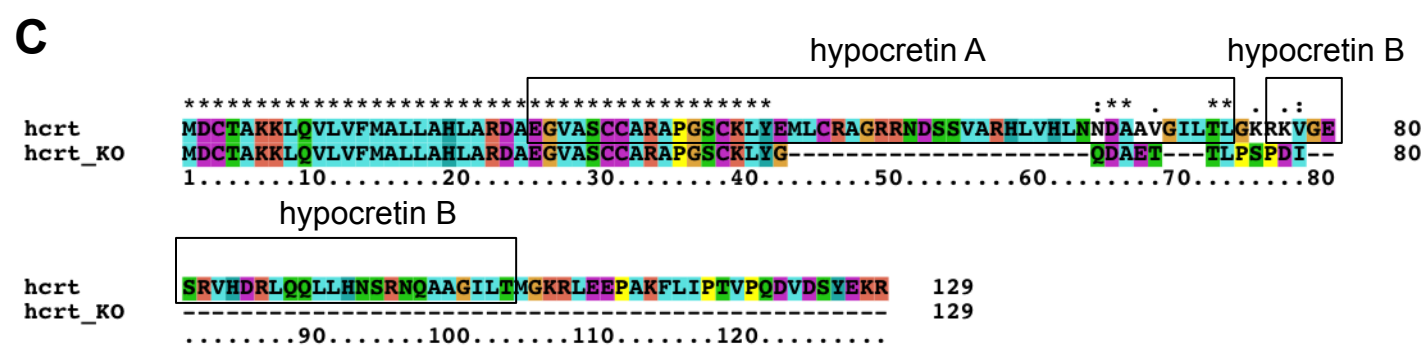

Figure S3 Generation of hcrt-KO zebrafish

Supplement: Supplementary file 8 [file Image_3.PDF]
