# Supplementary material for: Pharmacological profiling of zebrafish behavior using chemical and genetic classification of sleep-wake modifiers
Source: Front Pharmacol. 2015 Nov 3;6:257. doi: 10.3389/fphar.2015.00257 (PMC4630575; doi:10.3389/fphar.2015.00257)
Supplement: Supplementary file 7 [file Image_2.PDF]

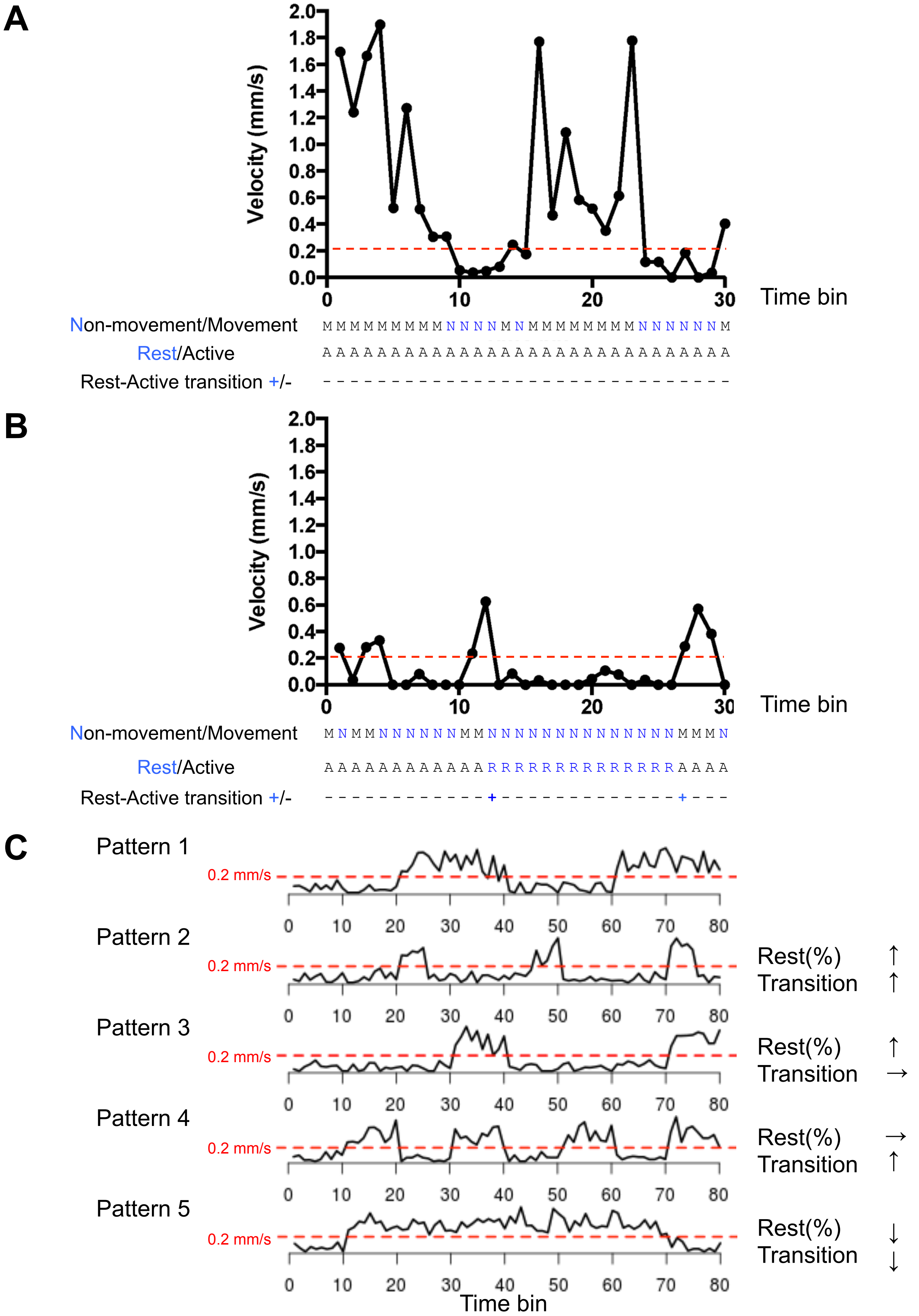

Figure S2 Measurements of rest state and transition between rest and active states
